# Evaluation of the physical activity level, nutrition quality, and depression in patients with metabolic syndrome: Comparative study: Erratum

**DOI:** 10.1097/MD.0000000000010886

**Published:** 2018-05-25

In the article, “Evaluation of the physical activity level, nutrition quality, and depression in patients with metabolic syndrome: Comparative study”,^[1]^ which appeared in Volume 97, Issue 18 of *Medicine*, the author affiliations appear incorrectly. They should appear as:

İsmet Kazaz, Msc^**a**^, Ender Angın, PhD^**a**^, Seray Kabaran, PhD^**b**^, Gözde İyigün, PhD^**a**^, Berkiye Kırmızıgil, PhD^**a**^, Mehtap Malkoç, PhD^**a**^

^a^ Eastern Mediterranean University, Faculty of Health Sciences, Physiotherapy and Rehabilitation Department; and ^b^ Eastern Mediterranean University, Faculty of Health Sciences, Nutrition and Dietetics Department, Famagusta, North Cyprus via Mersin 10, Turkey.

## References

[1] Kazaz İ Ender A Kabaran S Evaluation of the physical activity level, nutrition quality, and depression in patients with metabolic syndrome: Comparative study. *Medicine*. 2018 97:e10485. 10.1097/MD.0000000000010485 PMC6392583 29718839

